# *SPARC*-modified mesenchymal stem cells promote recovery of β-cells and insulin secretion by calcium ion homeostasis

**DOI:** 10.1186/s13287-025-04727-2

**Published:** 2025-11-03

**Authors:** Jiaqi Gao, Balun Li, Hongkai Tian, Chenchen Li, Nikita Merzlikin, Dongyao Han, Zixi Ling, Zengyu Zhang, Wenlong zhu, Jianqi Dai, Lydmila Gerunova, Changrong Lv, Na Li, Jinlian Hua

**Affiliations:** 1 College of Veterinary Medicine, Shaanxi Centre of Stem Cells Engineering & Technology, Northwest A&F University, Yangling, 712100 Shaanxi China; 2 Faculty of Veterinary Medicine, Omsk State Agrarian University, Omsk, 644122 Russia

**Keywords:** Diabetes, Mesenchymal stem cells, Ca^2+^, Islet beta cell, Glucose-stimulated Insulin Secretion

## Abstract

**Introduction:**

Type 1 diabetes (T1D) results from the destruction of pancreatic β-cells, leading to insulin deficiency. As insulin therapy does not affect disease progression, advancements in immune regulation therapies have emerged, including the reconstitution of the insulin secretory system. Cysteine-rich acidic secretory protein (*SPARC*) is an extracellular matrix glycoprotein that regulates cell adhesion, facilitating cell migration, and mediating interactions between cells and their extracellular matrix. *SPARC* is overexpressed during tissue repair and is involved in β-cells survival. However, the potential of *SPARC*-modified mesenchymal stem cells (MSCs) to improve insulin secretion has not been thoroughly investigated. This study investigated the therapeutic effects of *SPARC*-MSCs in vivo and in vitro and assessed whether *SPARC* enhances survival and insulin secretion after β-cells injury.

**Methods:**

In vivo, we established T1D models in mice and canine using *SPARC*-MSCs for cell transplantation. In vitro, MIN6 cells were damaged with STZ, and *SPARC*-MSC supernatant was co-cultured with MIN6 for various assays.

**Results:**

Our study demonstrated that *SPARC* enhanced the regenerative capacity and migratory efficiency of MSCs after H_2_O_2_ injury and improved their morphology. In STZ-induced canine and mice diabetes models, *SPARC*-MSCs therapy significantly reduced hyperglycemia, improved oral glucose tolerance test (OGTT), and reversed weight loss in canine. Biochemical analyses showed improved liver function, and histological examination revealed restored islet area was significantly restored. Transcriptome and proteome sequencing indicated significant enrichment in calcium binding and cell migration pathways. Co-culturing *SPARC*-MSC supernatant with MIN6 cells after STZ injury restored their regenerative ability, enhancing insulin secretion and ATP content under high glucose stimulation. *SPARC* treatment also significantly increased intracellular Ca^2+^ levels in MIN6 cells.

**Conclusion:**

*SPARC* significantly promotes cell regeneration and stimulates insulin secretion by increasing intracellular ATP and Ca^2+^ influx. In diabetic canine and mice models, it alleviated hyperglycemia, improved glucose tolerance, and enhanced pancreatic islet area and insulin secretion.

**Graphical Abstract:**

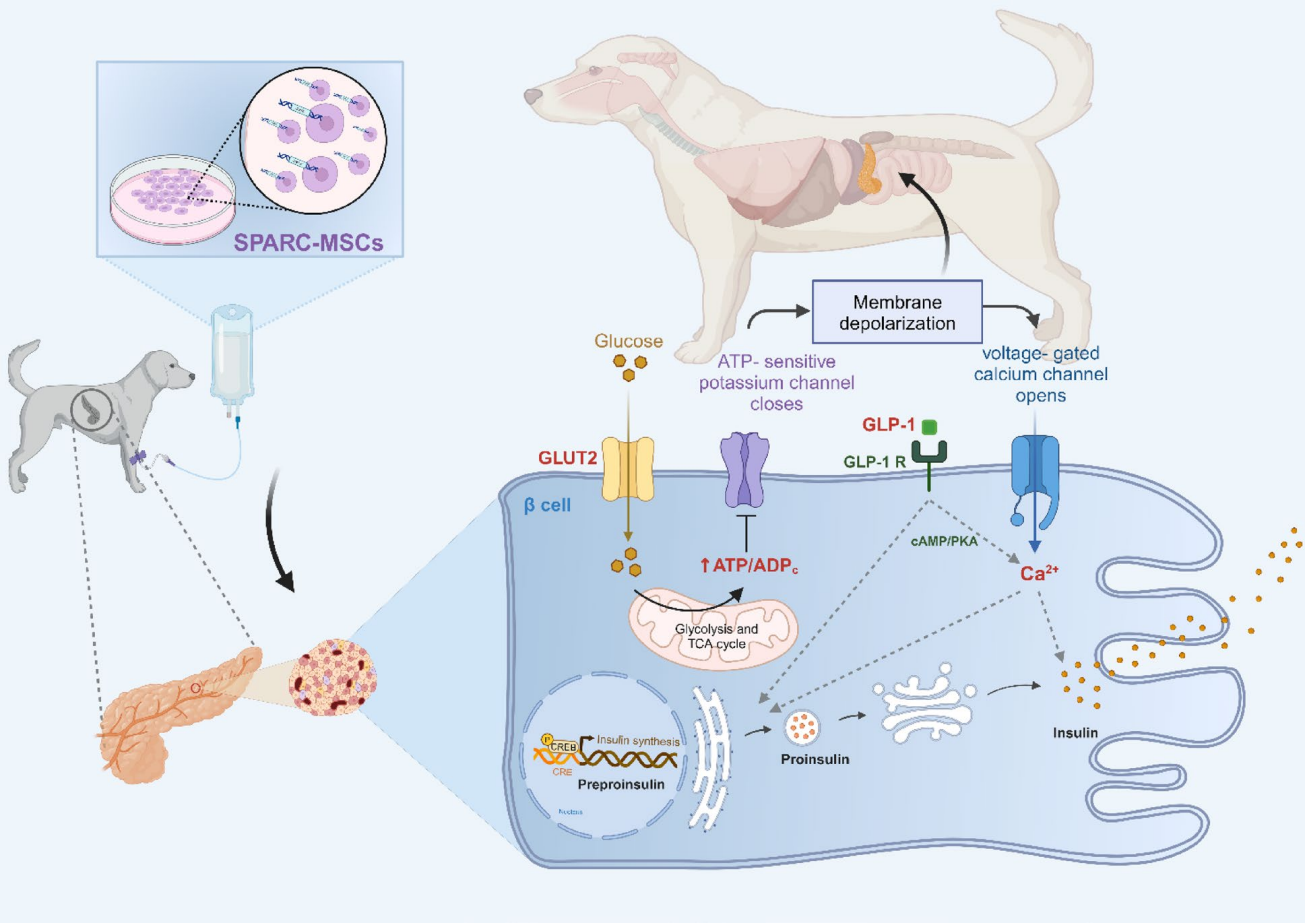

## Background

Diabetes mellitus is a metabolic disorder characterized by hyperglycemia, which arises from defects in insulin secretion, impaired insulin function or both. The overt symptoms of hyperglycemia include polyuria, polyphagia, and weight loss, while long-term complications encompass retinopathy, nephropathy, foot ulcers, and neuropathy [1]. Type 1 diabetes mellitus (T1DM) is an autoimmune disease that predominantly affects children and adolescents, leading to the destruction of pancreatic beta cells and resulting in an inability to produce insulin [2, 3]. However, treatment with insulin alone does not ameliorate the disease process, hyperglycemia, immune dysregulation, and inflammatory responses further damage pancreatic β-cells and activate various stress pathways, including oxidative stress, endoplasmic reticulum (ER) stress, mitochondrial dysfunction, apoptosis, and necrosis [4–6]. Consequently, therapeutic approaches that emphasize immunomodulation, such as restoring pancreatic β-cells function and re-establishing the insulin secretion system, may prove effective in preventing and reversing the progression of diabetes [7].

Mesenchymal stem cells (MSCs) exhibit several critical functions, including immunomodulation, the secretion of biomolecules such as growth factors and cytokines, evasion of innate immunity, antioxidant and anti-apoptosis [8]. Numerous studies have demonstrated the role of MSCs in diabetes, demonstrating their capacity to prevent β-cells destruction, safeguard the affected β-cells populations, and promote endogenous β-cells regeneration [9]. For instance, Bassi's team discovered that the transplantation of allogeneic MSCs into diabetic non-obese diabetic (NOD) mice significantly alleviates the symptoms of autoimmune diabetes, mitigates the Th1 immune response, and consequently supports the maintenance of functional β-cells [10]. In the context of islet transplantation for diabetes treatment, MSCs are capable of secreting a diverse array of factors, including growth factors and immunomodulators, which serve to protect islet cells and enhance graft survival [11–13]. Furthermore, exosomes derived from MSCs have been shown to ameliorate diabetes-induced muscle atrophy by promoting AMPK/ULK1-mediated autophagy [14].

Secreted protein acidic and rich in cysteine (*SPARC*) is an extracellular matrix glycoprotein that exhibits pleiotropic functions, including the regulation of cell adhesion, cell migration, growth factor activity, and the cell cycle. It mediates interactions between cells and their extracellular matrices and is known to be overexpressed during tissue renewal and repair following injury [15, 16]. Additionally, *SPARC* is implicated in various biological processes, such as obesity [17, 18], wound healing [19], inflammation [20], and cancers [21, 22], etc. Furthermore, *SPARC* plays a significant role in diabetes; research indicates that it promotes collagen formation and inhibits adipogenesis through the enhancement of beta-catenin signaling [17, 23]. Moreover, *SPARC* has been shown to regulate the survival of β-cells, with its overexpression in β-cells cultured under high glucose conditions resulting in increased insulin secretion [24, 25]. *SPARC* also can downregulate the RGS4 protein in pancreatic beta cells, further contributing to the increase in insulin secretion [26].

Insulin biosynthesis is regulated by multiple factors. When glucose enters β-cells, adenosine triphosphate (ATP) production increases via glycolysis and the tricarboxylic acid cycle (TCA). The elevated ATP/ADP ratio closes ATP-sensitive potassium channels (KATP), causing membrane depolarization. This triggers VDCC opening and subsequent Ca^2^⁺ influx, which is crucial for insulin secretion [27–31]. Additionally, *SPARC* has been implicated in the regulation of β-cells function and survival [24, 32], However, there is a lack of studies investigating the specific effects of *SPARC* on cell survival within pancreatic islet β-cells.

Canine diabetes mellitus (DM) is a prevalent spontaneous endocrine disorder in canines that exhibits clinical signs analogous to those of human T1DM. Although the pathogenesis of canines DM is unclear, both conditions necessitate lifelong administration of exogenous insulin to maintain glucose homeostasis [6]. As mammals, the pancreatic islets of canines share similarities with those of humans in terms of physiological status, size, and cellular composition [33], Consequently, the utilization of dogs as a translational model presents unique advantages, particularly given the increasing population of pet dogs that coexist in similar environments and routines with their owners.

In this study, we established canines and mice models of streptozotocin (STZ)-induced diabetes and administered *SPARC*-modified MSCs intravenously to assess their therapeutic effects. We found that in vivo administration of *SPARC* significantly ameliorated hyperglycemia, gradually restored STZ-induced islet damage, and facilitated the recovery of β-cells function and insulin production. Additionally, further in vitro experiments demonstrated that *SPARC* protected co-cultured MIN6 cells against STZ-induced apoptosis and enhanced glucose-stimulated insulin secretion (GSIS) by improving β-cells functionality through improving intracellular calcium ion levels and the ATP signaling pathway.

## Methods

### MSCs culture

MSCS and *SPARC*-MSCS cell lines were derived from previously established cell lines in the laboratory [34]. We acquired a 6-month-old female hybrid dog, weighing 1.5 kg, from the Experimental Animal Center of Northwest A&F University for the isolation of mesenchymal stem cells (MSCs). The mesenchymal stem cells were extracted from the subcutaneous adipose tissue of the dog's belly, with the comprehensive isolation protocols and validation outlined in our prior work [35]. The methodology we used for SPARC-modified MSCs was also detailed in our prior research [35]. Following thawing from liquid nitrogen tanks, the cells were cultured in α-MEM (Invitrogen, Carlsbad, CA, USA) complete medium, which was supplemented with 10% fetal bovine serum (FBS) (Bio-Channel, Nanjing) and penicillin/streptomycin, at 37 °C in a 5% CO2 incubator. The cells were passaged when the growth density reached approximately 80%. Cells that had undergone two passages following resuscitation were utilized for subsequent experiments.

### Assessment of the cytotoxic potency of H_2_O_2_

Cells from the MSCs and *SPARC*-MSCs groups were uniformly inoculated into 96-well plates containing culture medium (1.5 × 10^4^ cells/well, each concentration group contained 3 replicates) and incubated for 24 h after the addition of varying concentrations of H_2_O_2_ (0, 100, 200, 300, 400, 500, 600, and 700 µM). The CCK-8 assay was conducted by adding 10 µL of CCK-8 solution to each well and subsequently incubating the cells in a cell incubator for one hour. Absorbance was then measured at 450 nm using an ELISA Microplate reader.

### CCK-8 cell proliferation assay

The Cell Counting Kit-8 (CCK-8) assay was employed to evaluate the proliferative capacity of cells. MSCs and *SPARC*-MSCs group were uniformly inoculated into 96-well plates containing culture medium (1.5 × 10^4^ cells/well, each cell group contained 6 replicates), cultured with 200 µM H_2_O_2_ for 24 h, Subsequently, the medium was replaced with normal culture medium, and the cells were allowed to continue culturing for an additional 24 h. Cell viability was then assessed using the CCK-8 assay.

### Giemsa staining

MSCs and *SPARC*-MSCs were inoculated into 48-well plates containing culture medium (2 × 10^4^ cells/well). The cells were divided into 4 groups: MSCs-Giemsa, MSCs-Bright, *SPARC*-MSCs-Giemsa, and *SPARC*-MSCs-Bright, with 3 replicates each. After treatment with 200 µM H_2_O_2_ for 24 h, the medium was replaced with normal medium and maintained for an additional 24 h. The cells were fixed with 4% paraformaldehyde for 10 min at room temperature, rinsed three times with PBS, and stained with a prepared 1 × Giemsa staining solution for 15 min. After three additional rinses with PBS, the cells were examined and photographed under a light microscope in accordance with the instructions provided by the Giemsa staining kit (Beyotime).

### Cell scratch assay

MSCs and *SPARC*-MSCs were inoculated into 6-well plates containing medium (5 × 10^6^ cells/well, 3 replicates per group) and incubated with 200 µM H_2_O_2_ for 24 h. After discarding the medium, uniform scratches were made with a sterile gun tip, followed by washing with PBS and adding normal medium. Photographs were taken with a light microscope at 0, 6, 12, and 24 h after scratching.

### Animals

The work has been reported in line with the ARRIVE guidelines 2.0.

Thirty male ICR mice (26 ± 5 g) (6 weeks old) were purchased from Chengdu Dashuo Laboratory Animal Co. (Chengdu, China). The mice were provided with unrestricted access to distilled water and standard chow, they were maintained in an environment with a temperature between 24 and 26 °C, humidity levels ranged from 69 to 71%, and a 12:12 h light–dark cycle.

Fifteen healthy male hybrid canines (2–4 years old) were purchased from the Experimental Animal Centre of Northwest Agriculture and Forestry University (Xianyang, China). All canines were bred, obtained, and housed in accordance with the institutional guidelines for experimental animals. and received external and internal deworming treatment (Mopsion, ringpu). The canines were housed in cages within rearing rooms equipped with cleaning devices, where the temperature was maintained between 18 and 25 °C and humidity levels ranged from 40 to 60%. The lighting conditions were standard, and ambient noise levels were kept below 60 dB.

### Establishment and treatment of the STZ-induced diabetic animal model

After one week of acclimatization, mice were randomly divided into normal control and DM groups. The DM model was established via intraperitoneal injection of 40 mg/kg STZ (dissolved in pre-cooled sodium citrate buffer at pH 4.5) for 4 consecutive days. The normal control group (NC group, n = 6) received sodium citrate buffer. One week later, blood glucose levels were measured with a glucometer (ROCHE, Germany), and three consecutive readings above 16.7 mmol/L within one week were confirmed to DM model. The DM mice were randomly divided into the following three groups using a random number table, ensuring equal group sizes and no significant differences in baseline characteristics (n = 6 each): (a) DM group: received 0.2 ml saline intravenously; (b) MSCs group: diabetic mice received 1 × 10^6^ MSCs in 0.2 ml saline intravenously; (c) *SPARC*-MSCs group: diabetic mice received 1 × 10^6^ *SPARC*-MSCs in 0.2 ml saline intravenously. Injections were given biweekly for three cycles. Blood glucose and body weight were monitored weekly. The OGTT was performed to verify the treatment effect. At the end of the study, mice were fasted for 12 h, mice were deeply anesthetized with 1.2% tribromoethanol (0.1 ml/10 g, Nanjing Aibei Biotechnology, M2940). Then we made an abdominal midline incision and collect their pancreases. They were fixed in 4% neutral formalin for histopathological analysis. And then the rats were euthanized with carbon dioxide. The detailed procedure is shown in Fig. 3A

The method for establishing a DM canine model is basically similar to that utilized for mice models, with the exception that the STZ dosage is adjusted to 35 mg/kg. The criteria for the successful establishment of the model stipulate that fasting blood glucose remains above 11.1 mmol/L for two consecutive weeks, in conjunction with the presence of clinical signs such as polydipsia, polyphagia, polyuria, and weight loss. The canines were randomly divided into three groups using a random number table, ensuring equal group sizes and no significant differences in baseline characteristics (n = 3 each) and underwent a four-week treatment period. On the day of the trial's conclusion, the canines were fasted for 12 h. After measuring the OGTT, propofol (6 mg/Kg, Guangdong Jiabo Pharmaceutical) was administered through the cephalic vein to induce anesthesia. Once deep anesthesia was achieved, a rapid injection of 10% potassium chloride (DAMAO) was administered for euthanasia. The abdominal cavity was then opened along the median abdominal line, and pancreatic and hepatic tissues were collected and placed in a 4% formalin solution for fixation. The detailed procedure is shown in Fig. 2A.

### OGTT

Animals were fasted overnight (12 h, free water access). For the OGTT, glucose was administered orally at 2 g/kg for mice and 0.9 g/kg for canines. Blood samples were taken from the tail vein at 0, 15, 30, 60, 90, and 120 min and blood glucose levels were measured using a glucometer.

### Histological analysis

The pancreas and liver from each animal were fixed in 4% neutral formalin at 4 °C for 24 h, embedded in paraffin, sectioned to 5 μm, and stained with hematoxylin–eosin (H&E). The tissues were observed and photographed under a light microscope, and the images were analyzed with ImageJ software.

### Analysis of immunofluorescence staining

Paraffin sections of pancreatic tissue were baked at 65 °C for 30–60 min, dewaxed twice with xylene for 10 min each time, then immersed in a xylene-ethanol mixture. They were then sequentially treated with anhydrous ethanol and various concentrations of ethanol, followed by a wash with distilled water and Tris/EDTA buffer (pH 9.0). After Antigen retrieval, the sections were washed in PBST and incubated overnight at 4 °C with INS antibody (1:200, mice, proteintech), GLP-1 antibody (1:200, rabbit, proteintech), primary antibodies overnight at 4 °C. Afterward, they were treated with secondary antibodies at 37 °C for 1 h and stained with Hoechst 33342 (1:500, Sigma) for 5 min [36]. Finally, the sections were blocked with anti-fluorescent extractant and observed and photographed under a Laser Scanning Confocal Microscope (Leica, Germany). Images were further analyzed using ImageJ software.

### MIN6 cells culture

The mice islet tumor cell line (MIN6) (CL-0674) was purchased from Wuhan Procell. The MIN6 cell line was established from islet tumors of transgenic non-obese diabetic mice expressing the simian virus 40 large T antigen (controlled by the isletoxin promoter). The cells were cultured in RPMI 1640 medium supplemented (Corning, America) with 20% fetal bovine serum (Bio-Channel, Nanjing), penicillin/streptomycin, and 50 µm/L β-mercaptoethanol (Gibco, America) at 37 °C in a 5% CO2 incubator. Cells were passaged at a ratio of 1:2 when the growth density reached approximately 80%. For subsequent experiments, cells that had undergone two passages following resuscitation were utilized.

### Establishment of the STZ-induced MIN6 cell injury model, grouping, and co-culture methodology

MIN6 cells were uniformly inoculated into 96-well plates containing culture medium (1.5 × 10^4^ cells/well, each cell concentration set comprising 6 replicates. Following the addition of various concentrations of STZ (dissolved in ddH2O: 0, 5, 10, 15, 20, and 25 μM), the cells were cultured for 24 h. Cell viability was measured using the CCK-8 assay to determine the optimal injury concentration. The experimental procedure was performed as described in 2.3. MIN6 cells were categorized into 4 groups: (a) NC group: cells were cultured in standard medium; (b) DM group: following a 24 h STZ-induced injury, a co-culture was established with half of normal medium and half of the MIN6 cell supernatant for an additional 24 h; (c) MSCs group: following a 24 h STZ-induced injury, a co-culture was established with half of normal medium and half of MSCs supernatant for an additional 24 h; (d) *SPARC*-MSCs group: following a 24 h STZ-induced injury, a co-culture was established with half of normal medium and half of *SPARC*-MSCs supernatant for an additional 24 h.

### Survival of MIN6 cells following treatment after STZ-induced injury

Four groups of cells were inoculated into 96-well plates containing culture medium (1.5 × 10^4^ cells/well, 6 replicates per group). Following STZ-induced injury, the cells were treated, and cell survival was assessed using the CCK-8 assay.

### Glucose stimulated insulin secretion (GSIS) assay

The GSIS assay was used to evaluate the insulin secretion capacity of MIN6 cells. 4 groups of MIN6 cells were inoculated into 24-well plates (6 × 10^5^ cells/well, 3 replicates per group). Following STZ injury and treatment, the cells were initially washed with glucose-free Krebs–Ringer bicarbonate HEPES buffer (KRBH), then sugar-free KRBH buffer was added to each well, and the cells were incubated and starved for 1 h. After discarding the supernatant, the low-sugar KRBH buffer (2 mM) was added, and the cells were incubated for an additional 30 min. The supernatant was then collected. Following this, a high-sugar KRBH buffer (20 mM) was added, and the cells were incubated for another 30 min before collecting the supernatant. All collected supernatants were centrifuged at 1500 rpm for 15 min at 4 °C, and the insulin content in the cell supernatants was detected using an ultrasensitive ELISA kit (FANKEWEI, Shanghai, China).

### ELISA

Insulin concentrations in the supernatants of the four sets of cells, as described before were quantified using a double antibody sandwich assay with a 96 T ELISA kit for mice insulin (FANKEWEI, Shanghai, China). The assay was conducted in accordance with the manufacturer's instructions provided with the ELISA kit. For each group of cell supernatants, three duplicate wells were established. Absorbance was measured at 450 nm using an enzyme marker, and the insulin concentrations secreted by each group of cells were subsequently calculated based on the standard curve.

### ATP assays

Intracellular ATP content was measured using an enhanced ATP assay kit (meilunbio, Dalian, China), in accordance with the manufacturer's instructions. Briefly, cells were lysed with ATP lysis buffer and subsequently centrifuged at 12,000 g for 10 min at 4 °C. The supernatant was carefully removed and mixed with a dilution buffer containing luciferase. The relative light units were measured by multimode reader (Molecular Devices, LLC). A fresh standard curve was generated, and ATP content was calculated based on this curve.

### Measurement of cytosolic free calcium level (Ca^2+^)i

MIN6 cells were cultured in 96-well plates (1.5 × 10^4 cells/well, 6 replicates per group). The cells were subsequently incubated with 1 μM Fluo-4 AM (Beyotime) for 10 min at 20 °C in the dark. Following this incubation, the cells were washed and allowed to rest for 30 min to facilitate the de-esterification of the dye within the cytosol. Fluorescence intensity was then measured using a multimode reader (Molecular Devices, LLC). The relative fluorescence signals were measured at an excitation wavelength of 488 nm and an emission wavelength of 516 nm. The resulting was analyzed and shown as the area under the original curve (AUC), in accordance with previously described.

### Statistical analysis

All results were analyzed utilizing GraphPad Prism v9.0 software and expressed as mean ± SEM. The Shapiro–Wilk test was used to check if the data followed a normal distribution. Significant differences were determined using two-tailed unpaired Student’s t-tests for two-group comparisons and one- or two-way analysis of variance (ANOVA) with the Sidak’s test for multiple group comparisons. Spearman's correlation analysis was used for correlation analysis. P-values less than 0.05 were considered statistically significant.

## Results

### ***SPARC*** over-expression alleviates the detrimental effects of H_2_O_2_ on MSCs

**Fig. 1:**
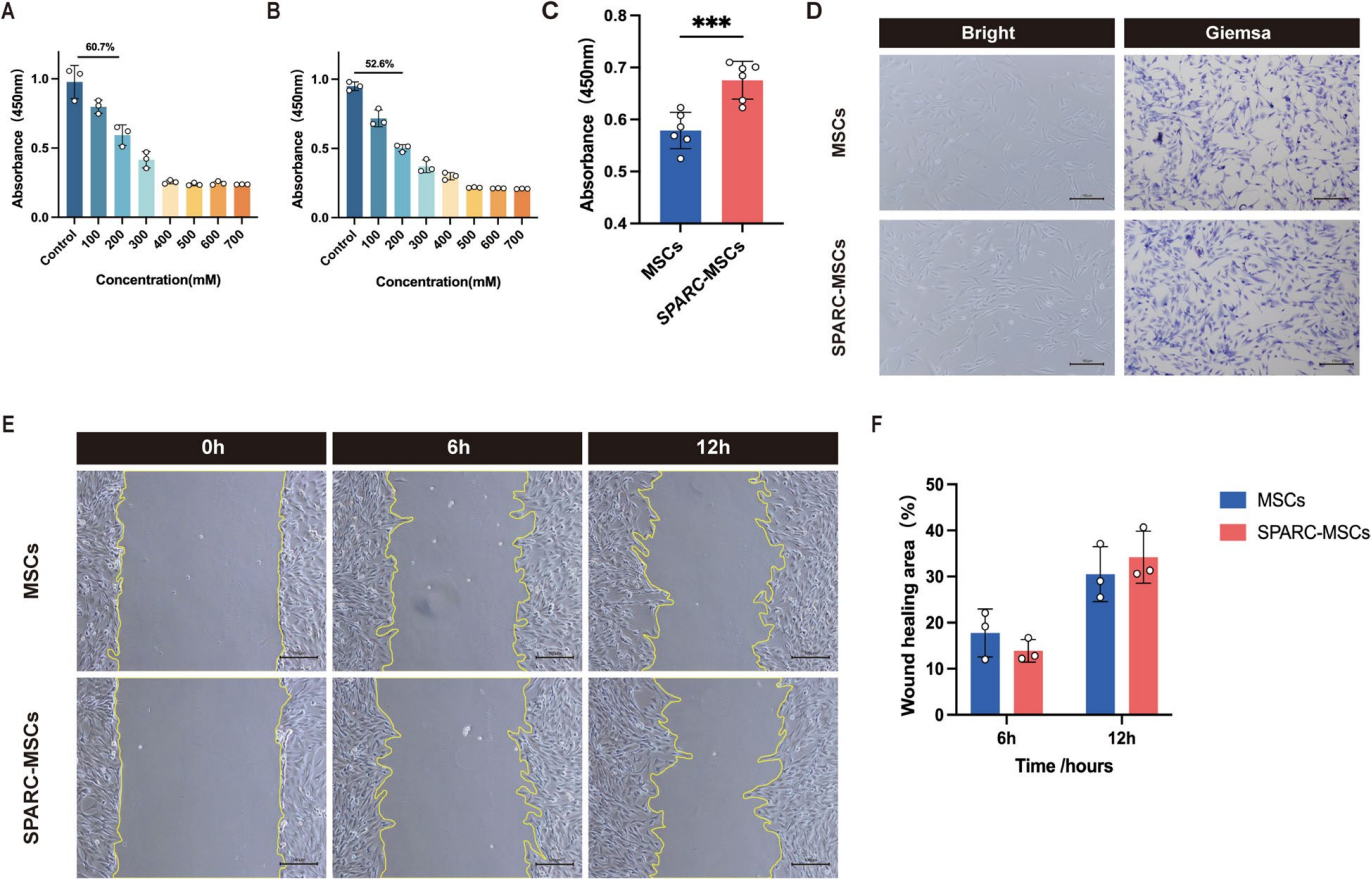
*SPARC* over-expression alleviates the detrimental effects of H_2_O_2_ on MSCs. **A**, **B** Screening of H_2_O_2_ damage concentration on *SPARC*-MSCs and MSCs. **C** Compared with MSCs group, *SPARC*-MSCs group effectively alleviated the proliferation ability of MSCs after H_2_O_2_ injury (n = 6 per group). **D** Cell morphology was significantly improved in *SPARC*-MSCs group compared with the MSCs group. Scale bar: 100 μm. **E**, **F** *SPARC*-MSCs group improved the migration ability of MSCs at 12 h. Scale bar: 100 μm. The results are presented as mean ± SEM. n = 3 per group unless noted. ***P < 0.001

To evaluate the adaptive regenerative capacity of *SPARC* following injury, MSCs and *SPARC*-MSCs were subjected to oxidative stress by exposure to 200 mM H_2_O_2_ for 24 h (Fig. 1A, B). Subsequently, the culture medium was replaced with normal medium, and the cells were cultured for an additional 24 h. The results obtained from the CCK8 assay indicated that the cell proliferation rate in the *SPARC*-MSCs group was significantly enhanced compared to the other group (Fig. 1C). Additionally, Giemsa staining revealed a restoration of cell morphology in the *SPARC*-MSCs group (Fig. 1D). Furthermore, the cell migration assay demonstrated that the *SPARC*-MSCs group exhibited a reversal of cell migration ability at the 12-h mark, compared to the 6-h assessment (Fig. 1E, F).

### *SPARC*-MSCs relieve hyperglycemia and increase islet area in canines with STZ-induced diabetes

**Fig. 2:**
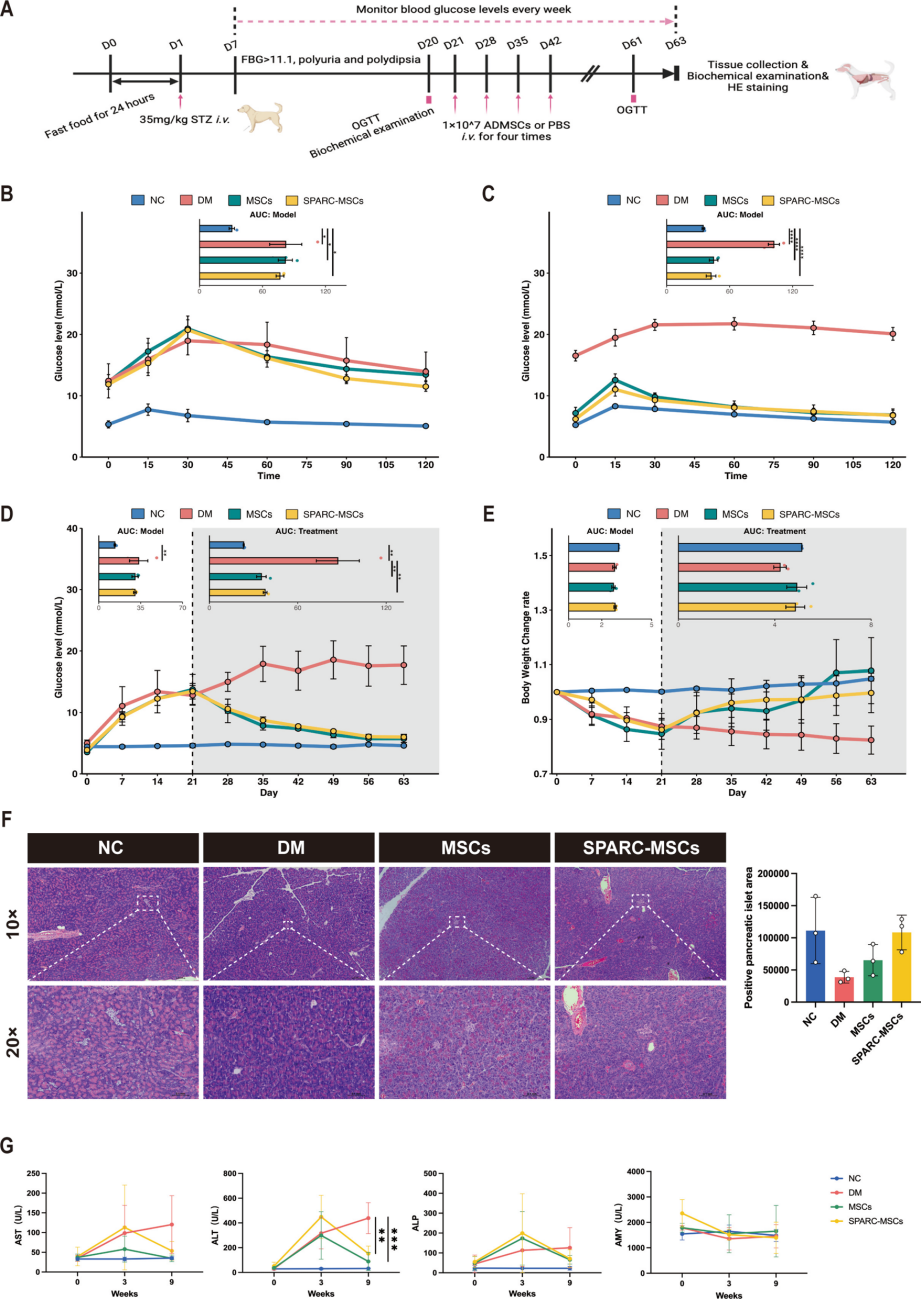
*SPARC*-MSCs relieve hyperglycemia and increase islet area in canines with STZ-induced diabetes. **A** Flowchart of MSC treatment for diabetic canines. STZ-induced diabetic canines were randomly divided into 3 groups: PBS-treated group (DM), MSC-treated group (DM + MSCs) and *SPARC*-MSCs-treated group (DM + *SPARC*-MSCs). Canines not injected with STZ served as the NC group. **B** OGTT validation after modelling in diabetic canines (**C**) 20 days after the fourth MSCs injection, glucose tolerance was significantly improved after *SPARC*-MSCs treatment compared to the DM group. **D** Blood glucose levels were improved after *SPARC*-MSCs treatment compared with DM group. **E** Improved body weight level after *SPARC*-MSCs treatment compared to the DM group. **F** HE staining of paraffin sections of canines pancreatic tissue and pancreatic islet area. Scale bar: 0.1 mm. **G** Trends of AST, ALT, ALP and AMY levels indices in canines serum biochemical tests at weeks 3 and 9 after STZ injection. The results are presented as mean ± SEM. n = 3 per group.* P* < 0.05, **P < 0.01, ***P < 0.001, ****P < 0.0001

We established a diabetic canine model through the intravenous injection of 35 mg/kg STZ, which was validated by monitoring blood glucose levels, body weight, and conducting an OGTT (Fig. 2A). Canines exhibiting weight loss and significantly elevated blood glucose levels in the STZ-injected group compared to the NC group. A notable decline in OGTT was recorded on day 20 after STZ-injection (Fig. 2B). The established diabetic canines (designated as DM) were randomly allocated into three groups (n = 3) and received intravenous injections of either 3 ml of saline, 1 × 10^7^ control MSCs or *SPARC*-MSCs suspended in 3 ml of saline, administered once weekly for four consecutive weeks. The saline-treated canines served as the control group. Blood glucose levels and body weight were monitored biweekly throughout the experimental period, and the OGTT was test on day 61 following STZ injection to assess glucose tolerance in the diabetic canines. In the *SPARC*-MSCs group, there was a significant reduction in blood glucose levels, restoration of body weight, and marked improvement in OGTT results compared to the DM group (Fig. 2C, D, E).

Biochemical analyses indicated that aspartate aminotransferase (AST), alanine aminotransferase (ALT), and alkaline phosphatase (ALP) levels were elevated during the third week following streptozotocin (STZ) injection, which was associated with mild liver injury. Notably, ALT levels exhibited a significant decrease by the ninth week after STZ injection, while no substantial changes were observed in AMY levels (Fig. 2G). HE staining of pancreatic tissue showed some recovery in the pancreatic islet area after treatment with *SPARC*-MSCs compared to the DM group. (Fig. 2F). These results suggest that *SPARC* are effective in ameliorating hyperglycemia and enhancing glucose tolerance in STZ-induced diabetic canines, as well as in partially restoring the pancreatic islet area.

### *SPARC*-MSCs improve glucose homeostasis and restore islet function in diabetic mice model

**Fig. 3:**
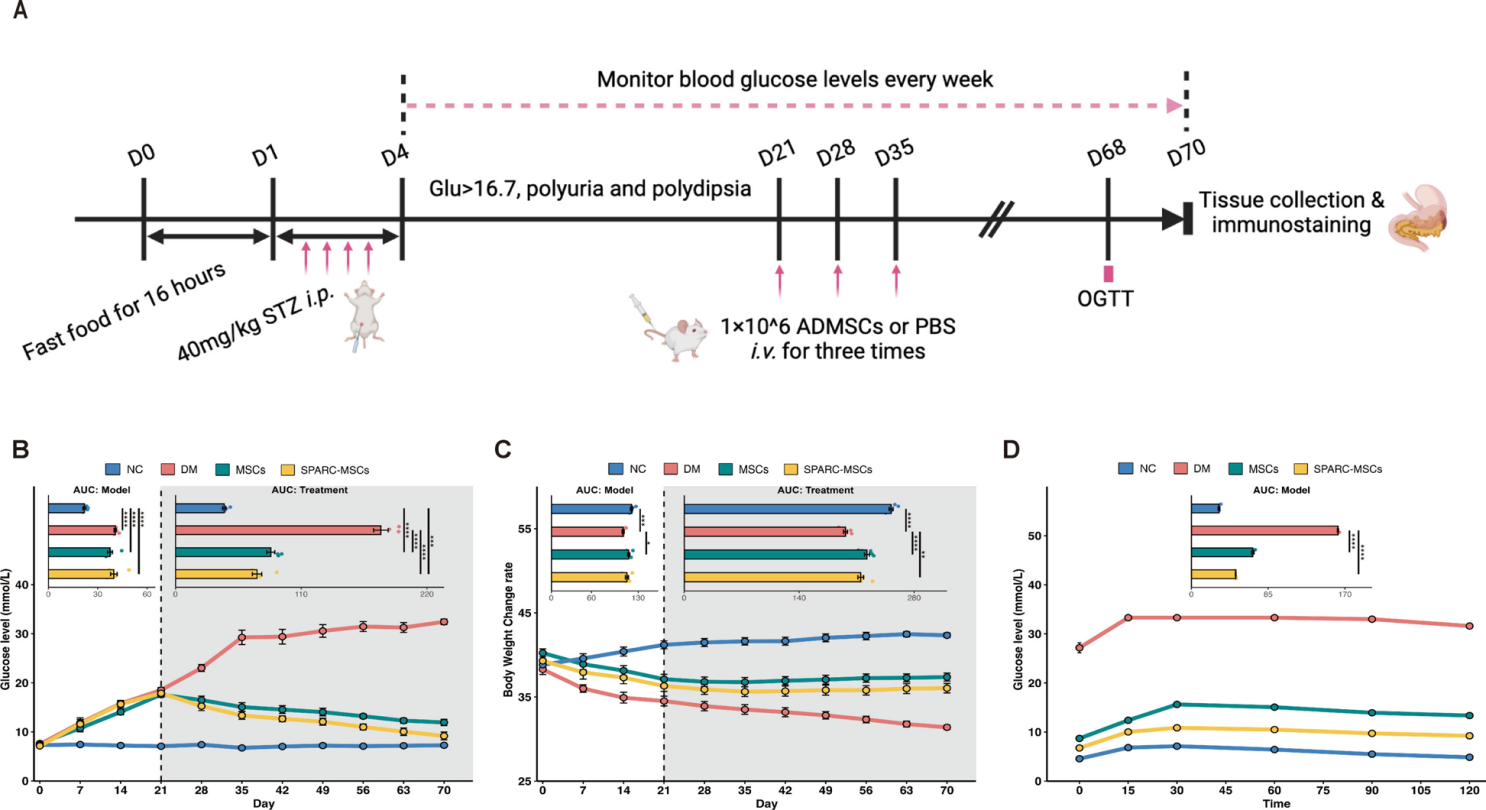
*SPARC*-MSCs improve glucose homeostasis and restore islet function in diabetic mice model. **A** Flowchart of MSCs treatment of diabetic mice. STZ-induced diabetic mice were randomly divided into 3 groups: PBS-treated group (DM), MSC-treated group (DM + MSCs) and *SPARC*-MSCs-treated group (DM + *SPARC*-MSCs). Mice not injected with STZ served as the NC group. **B** Blood glucose levels were improved after *SPARC*-MSCs treatment compared to the DM group. **C** Body weight level was improved after *SPARC*-MSCs treatment compared with DM group. **D** 47 days after the third injection of MSCs, glucose tolerance was significantly improved after treatment with *SPARC*-MSCs compared with the DM group (n = 3). The results are presented as mean ± SEM. unless otherwise stated, n = 6 per group. *P* < 0.01, ****P < 0.0001

We established a diabetic mice model through the intraperitoneal injection of 40 mg/kg STZ for 4 consecutive days. Blood glucose levels and body weight were continuously monitored (Fig. 3A). In comparison to the NC group, the STZ-injected group exhibited a reduction in body weight and a significant increase in blood glucose levels, with mice demonstrating random blood glucose levels ≥ 16.7 mmol/L on three consecutive times within one week (Fig. 3B). Diabetic mice (referred to as DM) were randomly divided into 3 groups (n = 6) on the 21st day after STZ injection. They were received intravenous injected of either 1 ml of saline or 1 × 10^6^ MSCs or *SPARC*-MSCs suspended in 1 ml of saline, administered once a week for 3 consecutive weeks. Mice treated with normal saline served as the NC group. Blood glucose levels and body weight were monitored weekly throughout the experimental period, and OGTT was test on day 68 following STZ injection to assess glucose tolerance in the diabetic mice (Fig. 3B, C, D). Compared to the DM group, treatment with *SPARC*-MSCs resulted in significant improvements in hyperglycemia, body weight, and OGTT outcomes in the diabetic mice.

**Fig. 4:**
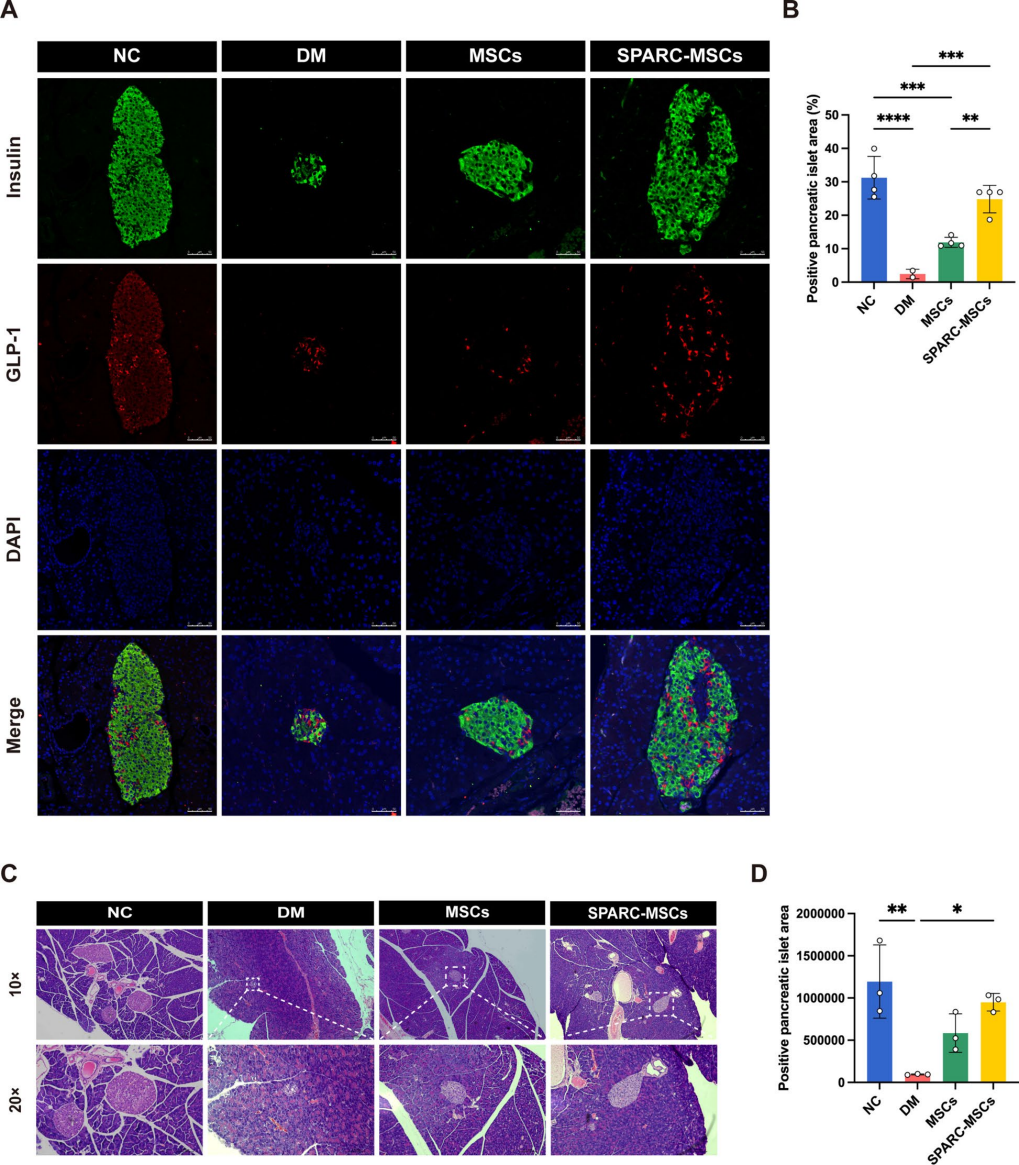
*SPARC*-MSCs improve glucose homeostasis and restore islet function in diabetic mice model. **A** Immunolocalization of insulin (green), GLP-1 (red) and DAPI (blue) in mice pancreatic tissue. Scale bar: 50 μm. **B** Pancreatic islet area and insulin secretion were significantly increased in the DM + *SPARC*-MSCs group compared to the DM group (n = 3) (**C**) HE staining of paraffin sections of mice pancreatic tissue. Scale bar: 0.1 mm. **D** The number and area of pancreatic islets in the pancreas were significantly increased in the DM + *SPARC*-MSCs group compared with the DM group (n = 4). The results are presented as mean ± SEM. *P* < 0.05, **P < 0.01, ***P < 0.001, ****P < 0.0001

STZ is a cytotoxic glucose analogue that is absorbed by pancreatic islet beta cells through the glucose transporter GLUT2. Upon entry into the cells, STZ inhibits DNA synthesis by inducing DNA fragmentation and methylation, which ultimately leads to the death of pancreatic islet β-cells. This process results in insufficient insulin secretion and the development of diabetes [37]. To assess islet function, we utilized immunofluorescence staining techniques. In comparison to the NC group, both the size of the islets and the quantity of pancreatic β-cells were significantly reduced in the DM group. After transplantation of *SPARC*-MSCs, a markedly enhanced immunostaining response for insulin and GLP-1 was observed, suggesting a significant recovery in both islet size and quantity, as well as an increase in the number of insulin-secreting pancreatic β-cells (Fig. 4A, B). HE staining of pancreatic tissue further demonstrated a significant recovery of pancreatic islet area after treatment with *SPARC*-MSCs when compared to the DM group (see Fig. 4C, D). These findings indicate that the transplantation of *SPARC*-MSCs not only improved glucose homeostasis but also effectively reversed apoptosis of pancreatic β-cells and restored islet function in mice with STZ-induced diabetes.

### *SPARC* promote calcium ion influx to restore insulin secretion in MIN6 cells.

**Fig. 5:**
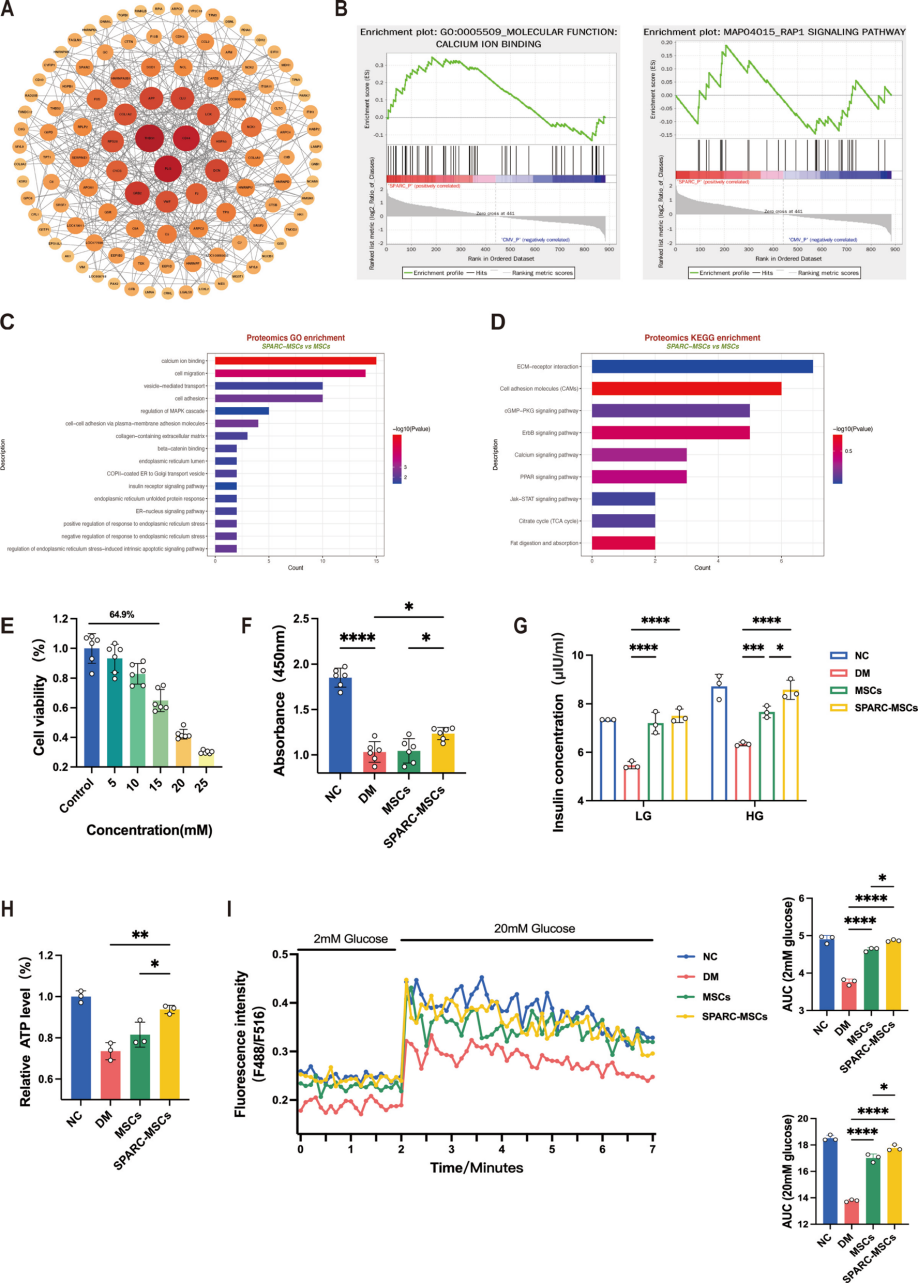
*SPARC* promote calcium ion influx to restore insulin secretion in MIN6 cells. **A** Transcriptomic differential gene analysis of *SPARC*-MSCs and MSCs. **B** Proteomic GSEA analysis of *SPARC*-MSCs and MSCs. **C** Proteomic GO analysis of *SPARC*-MSCs and MSCs. **D** Proteomic KEGG analysis of *SPARC*-MSCs and MSCs. **E** Screening of the optimal concentration of STZ-injured MIN6 cells. **F** Compared with the DM group, treatment with *SPARC*-MSC supernatant significantly improved the proliferation of injured MIN6 cells. **G** GSIS assay was performed on the treated MIN6 cells after injury using 2 mM and 20 mM glucose, respectively, and *SPARC*-MSCs significantly improved the insulin secretion of MIN6 cells compared to the DM group. **H** Compared with the DM group, *SPARC*-MSCs significantly improved the intracellular ATP content. **I** Measurement of calcium ion content in MIN6 cells at 2 mM and 20 mM glucose concentrations

To elucidate the mechanism of action of *SPARC*, we conducted proteomic sequencing on *SPARC*-MSCs and MSCs. As illustrated in Fig. 5A, B, C and D, the proteomic analysis revealed that a total of 216 proteins were upregulated, while 163 proteins were downregulated in the *SPARC*-MSCs group in comparison to the MSCs group. Furthermore, gene set enrichment analysis (GSEA), Gene Ontology (GO) enrichment analysis, and Kyoto Encyclopedia of Genes and Genomes (KEGG) enrichment analysis indicated that the *SPARC*-MSCs group exhibited significant enrichment in pathways related to calcium binding, cell migration, and endoplasmic reticulum stress.

Then we aimed to investigate the capacity of *SPARC* to facilitate the repair of MIN6 cells following injury. Initially, we assessed the impact of varying concentrations of STZ on MIN6 cell viability, determining that the survival rate of MIN6 cells exposed to 15 mM STZ was 64.9% (Fig. 5E). Consequently, all subsequent experiments utilized 15 mM STZ to induce injury in MIN6 cells. As illustrated in Fig. 5F, treatment of the damaged MIN6 cells with *SPARC*-MSC supernatant resulted in a significant enhancement of cellular repair after injury. To further evaluate whether *SPARC* could improve insulin secretion from the injured MIN6 cells, we performed the GSIS. The results indicated that both the MSC and *SPARC*-MSC groups significantly increased insulin release following treatment when stimulated with 2 mM and 20 mM glucose, in comparison to the DM group. Notably, under 20 mM glucose stimulation, the *SPARC*-MSCs group exhibited a significantly greater stimulation of insulin secretion than the MSCs group (Fig. 5G).

In general, upon the entry of glucose into β-cells, ATP is synthesized in the mitochondria through the processes of glycolysis and the tricarboxylic acid (TCA) cycle. An increase in the relative ratio of ATP to ADP results in the closure of ATP-sensitive potassium channels, leading to membrane depolarization. This depolarization subsequently stimulates the opening of voltage-gated calcium channels and promotes the release of calcium from the intracellular endoplasmic reticulum, thereby enhancing both insulin secretion and mitochondrial oxidative phosphorylation. The influx of intracellular calcium is a critical factor in the regulation of insulin release from pancreatic β-cells [37]. To further investigate whether *SPARC* can stimulate insulin release by promoting calcium ion influx, we first assessed the intracellular ATP content, as illustrated in Fig. 5H. Four groups of cells, namely NC, DM, MSCs and *SPARC*-MSCs, were subjected to injury, treatment, and stimulation with 20 mM glucose for 30 min. The cells were subsequently collected using ATP lysis buffer, and the ATP content was quantified using an enhanced ATP assay kit. The results indicated that the ATP content in the *SPARC*-MSCs group was significantly higher than that in the DM and MSCs groups. Given that intracellular calcium influx is pivotal in the regulation of insulin release from pancreatic β-cells, we measured the intracellular calcium ion concentration using the calcium ion probe Fluo-4. To evaluate whether *SPARC* promotes GSIS via intracellular calcium concentration ((Ca^2+^)i) in β-cells, we assessed (Ca^2+^)i in MIN6 cells using a multimode microplate reader. As illustrated in Fig. 5I, (Ca^2+^)i was increased at 20 mmol/L glucose compared to 2 mmol/L glucose in the *SPARC* group. Conversely, (Ca^2+^)i was inhibited in STZ group. These results suggest that *SPARC* facilitates the recovery of MIN6 cells from injury and enhances GSIS by stimulating inward calcium flux, which in turn generates ATP.

## Discussion

T1DM is a metabolic disorder characterized by the destruction of pancreatic β-cells, leading to absolute insulin deficiency and consequently elevated blood glucose levels [1]. Persistent hyperglycemia is associated with a range of complications, including retinopathy, which may result in vision loss [38]; nephropathy, which can lead to renal failure [39]; peripheral neuropathy, which may cause foot ulcers or necessitate amputation [40]; and autonomic neuropathy, which can give rise to gastrointestinal and genitourinary issues, cardiovascular symptoms, and other health conditions [41]. These complications significantly diminish the quality of life for affected individuals.

The pathogenesis of T1DM is intricately associated with both genetic and environmental factors. Key contributors to T1DM include β-cell stress, inflammatory responses, and various environmental influences. These pathological alterations culminate in an autoimmune response. The interactions between T cell receptors (TCR), peptides, and major histocompatibility complex (MHC) molecules within pancreatic islet cells facilitate the activation of adaptive immunity. CD4 + T cells are categorized into T helper cell type 1 (Th1) and type 2 (Th2) subsets. Th1 cells secrete pro-inflammatory cytokines, such as interleukin-2 (IL-2) and interferon-gamma (IFN-γ), which in turn activate CD8 + T cells and B cells, thereby contributing to the pathological mechanisms underlying T1DM. CD8 + T cells can directly target and attack β-cells through the Fas/FasL signaling pathway, which is crucial for maintaining peripheral self-tolerance and represents an initial step in apoptotic signaling. Prolonged exposure to stress-related factors, including excessive β-cell stress, free radicals, hyperglycemia, and IL-1β produced by host pancreatic immune cells, results in increased expression of Fas on the surface of β-cells. The recognition of β-cells by activated self-reactive T lymphocytes expressing FasL leads to the formation of the death-inducing signaling complex (DISC) effector complex, ultimately activating caspase-8 and inducing apoptosis in β-cells. Consequently, reliance solely on exogenous insulin can only mitigate symptoms; however, persistent hyperglycemia and inflammatory responses will further compromise pancreatic β-cells and potentially affect the entire pancreas, resulting in irreversible complications [2]. Therefore, there is an urgent need for therapeutic strategies that emphasize immunomodulation, including the restoration of pancreatic β-cell function and the reconstruction of the insulin secretion system.

MSCs are abundantly sourced from various tissues and can be efficiently expanded in vitro. They have demonstrated therapeutic potential by facilitating tissue repair, evading innate immune responses, and releasing biomolecules, including soluble cytokines and growth factors, which contribute to their immunomodulatory functions. Prior research has indicated that *SPARC* regulates several cellular processes, including cell adhesion, migration, growth factor activity, and the cell cycle. Additionally, *SPARC* mediates interactions between cells and their extracellular matrix and is known to be overexpressed during tissue renewal and repair following injury. Consequently, the objective of the present study was to investigate the therapeutic effects of *SPARC*-modified MSCs in the context of T1DM.

As companion animals, dogs inhabit a living environment, routine, and diet that closely resemble those of humans. Furthermore, as mammals, the canines pancreas exhibits similarities in function, size, and cellular composition to the human pancreas. The non-obese diabetic (NOD) mouse serves as an exemplary animal model for human T1DM, as it spontaneously develops tolerance to islet autoantigens early in life. Prior to the infiltration of lymphocytes, the pancreas of NOD mice demonstrates abnormal islet physiology, which includes vasculopathy, increased endoplasmic reticulum (ER) stress, and increased expression of inflammatory cytokines. These pathological changes can lead to β-cell dysfunction and subsequent cell death, resulting in the release of self-antigens and the activation of specific autoreactive T cells. Consequently, the present study aims to investigate the therapeutic effects of *SPARC*-modified MSCs using canines and mice animal models of diabetes that have undergone STZ injury.

In our study, we observed that *SPARC* enhanced the regenerative capacity of MSCs following H_2_O_2_ injury, as evidenced by improved migratory efficiency at 12 h and favorable alterations in cellular morphology. In a STZ-induced canine model of diabetes mellitus (DM), cell therapy with *SPARC*-MSCs significantly alleviated hyperglycemia and improved OGTT results, while also mitigating the weight loss observed in affected canines. Biochemical analyses indicated a marked improvement in liver function post-treatment. HE staining of pancreatic tissue sections revealed a significant restoration of islet area following *SPARC* treatment. To further investigate the mechanisms underlying the amelioration of diabetes by *SPARC*, we conducted cell therapy in STZ-induced diabetic mice. The results demonstrated that *SPARC* significantly reduced blood glucose levels and improved OGTT outcomes in these mice. Additionally, immunofluorescence staining of pancreatic tissue sections indicated a substantial increase in insulin secretion and a notable restoration of islet area post-treatment, findings that were corroborated by HE staining.

Insulin secretion is intricately associated with the homeostasis of (Ca^2+^)i. Following the entry of glucose into β-cells, mitochondria generate ATP through glycolysis and the tricarboxylic acid (TCA) cycle. An increase in the ATP to ADP ratio leads to the closure of ATP-sensitive potassium channels, resulting in membrane depolarization. The stimulation of the endoplasmic reticulum by intracellular calcium ions further promotes insulin secretion and mitochondrial oxidative phosphorylation. Consequently, we aimed to investigate whether *SPARC* could enhance insulin secretion by modulating Ca^2+^ homeostasis. Initially, we sequenced the transcriptome and proteome of *SPARC*-MSCs and MSCs. The results indicated a significant enrichment of *SPARC* in pathways related to calcium binding and cell migration. Subsequently, we observed that *SPARC* significantly restored the regenerative capacity of MIN6 cells following injury when co-cultured with the supernatant of *SPARC*-MSCs after STZ injury. Furthermore, *SPARC* markedly improved insulin secretion and ATP levels under high glucose stimulation in GSIS. Our findings regarding intracellular calcium ion content demonstrated that *SPARC* treatment also significantly elevated (Ca^2+^)i levels in MIN6 cells.

This work demonstrated the therapeutic effectiveness of *SPARC*-MSCs in murine and canine models, nevertheless two significant limitations must be acknowledged. The canine cohort had just three animals per group, potentially diminishing statistical power for identifying minor treatment benefits. Subsequent studies with higher sample sizes (n ≥ 6 per group) are necessary to validate repeatability. Subsequently, systemic intravenous administration, while clinically viable, demonstrated restricted pancreatic targeting efficacy. Creating tailored delivery methods, such as intra-arterial infusion or modified MSC membranes containing pancreatic homing peptides and exosomes-small extracellular vesicles secreted by MSCs may improve treatment precision..

## Conclusions

In summary, our study provides evidence that *SPARC* plays a crucial role in promoting cell regeneration following injury and enhancing insulin secretion through elevated intracellular ATP levels and Ca^2+^ influx. In diabetic canine and mouse models, *SPARC* significantly reduced hyperglycemia, improved glucose tolerance, and markedly increased both pancreatic islet area and insulin secretion.

## Data Availability

Not applicable.
